# Hepatitis C virus infection among HIV-positive men who have sex with men: protocol for a systematic review and meta-analysis

**DOI:** 10.1186/2046-4053-3-31

**Published:** 2014-03-26

**Authors:** Holly Hagan, Joshua Neurer, Ashly E Jordan, Don C Des Jarlais, Jennifer Wu, Kirk Dombrowski, Bilal Khan, Ronald Scott Braithwaite, Jason Kessler

**Affiliations:** 1 College of Nursing, New York University, New York, USA; 2 School of Social Work, New York University, New York, USA; 3 Beth Israel Medical Center, New York, USA; 4 School of Medicine, New York University, New York, USA; 5 Department of Sociology, University of Nebraska, Lincoln, USA; 6 John Jay College of Criminal Justice, New York, USA

**Keywords:** Hepatitis C virus, Men who have sex with men, HIV infection, Epidemiology, Systematic review

## Abstract

**Background:**

Outbreaks of hepatitis C virus (HCV) infection have been reported in HIV-positive men who have sex with men (MSM) in North America, Europe and Asia. Transmission is believed to be the result of exposure to blood during sexual contact. In those infected with HIV, acute HCV infection is more likely to become chronic, treatment for both HIV and HCV is more complicated and HCV disease progression may be accelerated. There is a need for systematic reviews and meta-analyses to synthesize the epidemiology, prevention and methods to control HCV infection in this population.

**Methods/design:**

Eligible studies will include quantitative empirical data related to sexual transmission of HCV in HIV-positive MSM, including data describing incidence or prevalence, and associations between risk factors or interventions and the occurrence or progression of HCV disease. Care will be taken to ensure that HCV transmission related to injection drug use is excluded from the incidence estimates. Scientific databases will be searched using a comprehensive search strategy. Proceedings of scientific conferences, reference lists and personal files will also be searched. Quality ratings will be assigned to each eligible report using the Newcastle–Ottawa scale. Pooled estimates of incidence rates and measures of association will be calculated using random effects models. Heterogeneity will be assessed at each stage of data synthesis.

**Discussion:**

HIV-positive MSM are a key HCV-affected population in the US and other high-income countries. This review seeks to identify modifiable risk factors and settings that will be the target of interventions, and will consider how to constitute a portfolio of interventions to deliver the greatest health benefit. This question must be considered in relation to the magnitude of HCV infection and its consequences in other key affected populations, namely, young prescription opioid users who have transitioned to illicit opiate injection, and older injection drug users among whom HCV prevalence and incidence are extremely high. This review is part of a series of systematic reviews and meta-analyses that will synthesize the evidence across all these population groups and develop recommendations and decision tools to guide public health resource allocation.

**Trial registration:**

PROSPERO registration number: CRD42013006462

## Background

### Introduction

There are an estimated 8.4 million men who have sex with men (MSM) in the US [1]; 16% are HIV positive [2]. Since 2000, there have been multiple reports of outbreaks of sexually transmitted acute hepatitis C virus (HCV) infection in HIV-positive MSM in the US, Canada, Europe, Japan and Australia [3]. The evidence points to blood as the medium of HCV exposure in sexual transmission [4,5]. A recurring context for transmission is group sex settings, where multiple individual risks converge including serosorting on the basis of HIV-positive status and sexual practices that cause trauma to mucosal surfaces and rectal bleeding [6-9].

In HIV-infected patients, acute HCV infection is more likely to become persistent, and HCV treatment is less likely to result in cure [10]. The risk of advanced liver fibrosis is elevated twofold in co-infected patients and HIV treatment is complicated in the presence of HCV-related liver damage [11]. Liver disease and hepatocellular carcinoma have become leading causes of death in HIV-infected individuals [12], and the risk of hepatocellular carcinoma is three to eight times higher among co-infected vs HCV mono-infected patients [13]. In general, HIV/HCV co-infection is characterized by decreased response to therapies and increased rates of both HIV and HCV disease progression [14].

Current recommendations to prevent HCV in HIV-positive MSM center on detecting acute infection through screening for elevated liver enzymes every three months and providing immediate HCV treatment to HCV RNA positive patients to prevent chronic HCV infection [10,15]. Early treatment of acute HCV infection in HIV-positive patients is associated with high response rates [16]. New direct-acting antiviral treatments for HCV infection have been shown to increase the likelihood of cure, and the course of treatment may be substantially shorter than with older regimens [17]. However, high rates of reinfection post-HCV treatment in HIV-positive MSM have been reported [18], and the cost of treatment, particularly with the new direct-acting antivirals, may limit the feasibility of this approach to HCV control [19].

**Figure 1:**
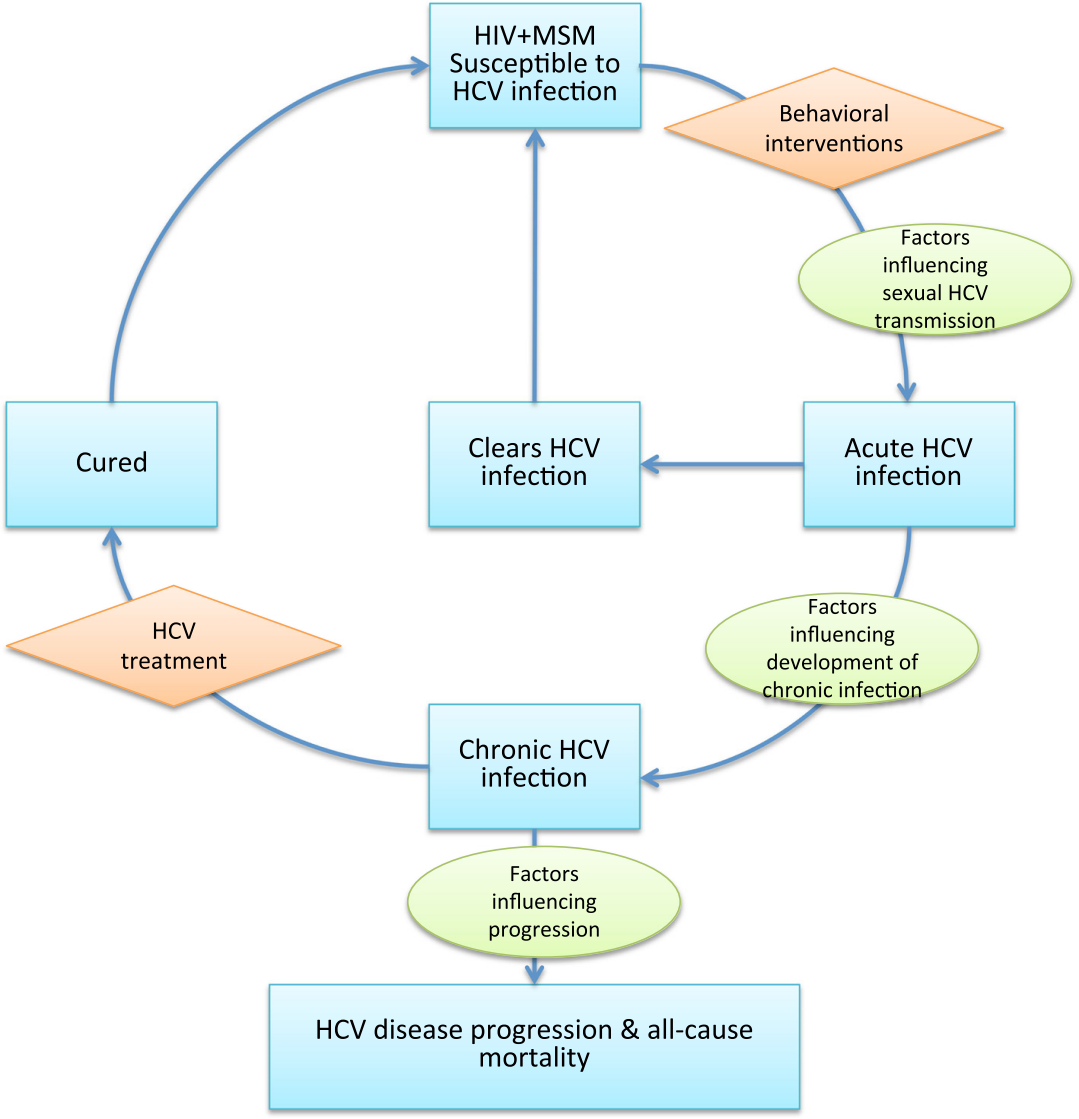
**Scope of the systematic review.** The figure shows the disease state (blue rectangles), interventions (orange diamonds) and factors influencing state transitions (green ovals) in sexual HCV transmission and natural history of HIV-positive men who have sex with men. HCV, hepatitis C virus; MSM, men who have sex with men.

In this systematic review, we will synthesize existing data describing rates of sexual HCV transmission in HIV-positive MSM and factors associated with transmission, rates of reinfection post-HCV treatment and the effects of interventions to prevent or treat HCV infection. Figure 1 shows the logic model guiding this review. Estimates derived from the review will be used to inform simulations of the effect (and the cost) of early treatment and behavioral and other interventions on HCV transmission and natural history in HIV-positive MSM. This systematic review and meta-analysis and related modeling are part of the HCV Synthesis Project, which is funded to develop guidance and recommendations for HCV control strategies for the United States.

### Previous reviews

HCV infection related to drug use has been addressed in several previous systematic reviews. These have covered HCV epidemiology in people who smoke or inhale drugs such as heroin, cocaine or crack [20], HCV epidemiology in people who inject drugs (PWID) [21,22], prevention and treatment of HCV infection in PWID [23-26], and heterosexual HCV transmission [27]. We are aware of only one systematic review describing HCV incidence in HIV-positive MSM; this review included 12 eligible articles [28]. HCV incidence rates among HIV-positive MSM ranged from 0 to 41.7/1,000 person years (PY), with a pooled rate of 6.08/1,000 PY (95% CI 5.18 to 6.99). A limitation of this review was that it did not describe a method to screen or classify studies in relation to sexual vs drug-related routes of exposure to HCV. Indeed, the inadvertent inclusion of HIV-positive MSM injectors may overestimate HCV seroconversion rates, because parenteral exposure is a much more efficient route of HCV transmission [29]. We believe that there have been no previous reviews synthesizing the risk of reinfection post-HCV treatment, which is highly relevant to the design of HCV control programs. Thus, the systematic review proposed here will make a unique contribution to our understanding of sexual HCV transmission, its consequences and control in HIV-positive MSM.

## Methods/design

### Design and scope

This study will consist of a systematic review and meta-analysis of the epidemiology, natural history and methods to control sexually transmitted HCV infection in HIV-positive MSM, as shown in Figure 1. Reports published or available from 1 January 1990 (when HCV testing became available) through 31 December 2013 will be included.

### Criteria for considering studies

#### *Inclusion and exclusion criteria*

We will include published and unpublished quantitative epidemiologic and intervention studies using cross-sectional, cohort (prospective and retrospective) and case–control designs. Epidemiologic studies will report HCV prevalence, incidence or risk factor analyses. Intervention studies will report on the effects of interventions to prevent or treat HCV infection in this population. Studies must include male individuals who are HIV-positive and who reported having sex with other men. We will not include data related to HIV-negative MSM or heterosexual men. An explicit mention that HIV-positive MSM using injection drugs were excluded from the analysis will be required because such individuals likely acquired HCV infection through the use of contaminated injection equipment rather than from sexual exposure. In studies that included both injecting and non-injecting MSM, separate estimates must be provided for non-injectors.

Studies among incarcerated persons will also be excluded, as infection rates and circumstances surrounding transmission are not likely to be generalizable to the broader population of HIV-positive MSM. Epidemiologic studies that enrolled HIV-positive MSM from settings where infection, exposure or disease progression rates were likely to be biased, such as in hepatology or gastroenterology clinics, or in sexually transmitted infection clinics, will be included but this will be noted in the quality ratings. All ages and individuals from any racial or ethnic group will be included. The search will include English-language reports available from 1 January 1990 through 31 December 2013.

#### *Outcome measures*

All reports must state that laboratory tests were used to ascertain HCV infection among subjects. Data reports that used self-reports or questionnaires to identify HCV status will be excluded. Rates of acute HCV infection or seroconversion, and reinfection with HCV post-HCV treatment in HIV-positive MSM will be included. We define subjects with ‘HCV seroconversion’ or ‘acute HCV’ as those who have been screened and tested positive for HCV antibodies (serology) or RNA within 12 months of previously testing negative. We adopted the European AIDS Treatment Network (European NEAT) Acute Hepatitis C Infection Consensus Panel criteria [30].

The *preferred criteria* are seroconversion or positive HCV RNA and a documented negative HCV RNA or negative HCV antibody in the previous 12 months. *Alternative criteria* include positive HCV RNA and an elevated ALT with or without other clinical signs of hepatitis.

Studies using the preferred criteria will be given higher quality ratings than those that use the alternative criteria. HCV reinfection will be defined as newly detectable HCV RNA following clearance of the infection in response to treatment of HCV infection.

#### *Exposure measures*

Exposures of interest include sexual practices that are hypothesized to increase the risk of blood exposure and thereby facilitate HCV transmission. These include unprotected receptive and insertive anal sex, practices that can damage the genital and anal mucosal surfaces such as fisting or ‘rough sex’, participation in group sex events and prolonged periods of sexual activity [8]. Age, race/ethnicity, calendar time, geographic location, substance use and other characteristics of the study sample will be collected as potential confounders or modifiers of the influence of exposures on HCV infection. Interventions of interest include behavioral interventions to reduce sexual risk behavior, condom distribution programs, liver enzyme screening and early treatment, and others that may emerge.

### Search strategy

The search strategy was developed in consultation with a medical librarian. The PubMed, EMBASE and BIOSIS databases will be searched using terms that cover the themes hepatitis C virus, men who have sex with men, transmission, prevention and treatment. Additional reports will be located by searching conference proceedings (e.g., the American Association for the Study of Liver Diseases, European Association for the Study of the Liver, International Conference on HIV/AIDS and Conference on Retroviruses and Opportunistic Infections), investigators’ personal files, and reference lists of reviews and related articles.

#### *Screening and data collection*

Reports obtained via the search strategy (abstracts and full-text articles) will be imported into Endnote X6 [31] and duplicates will be deleted. Reasons for exclusion will be recorded. Relevant data will be abstracted onto a paper instrument adapted from those used in a series of prior systematic reviews of HCV infection led by the first author [32]. Once this is complete, the data will be entered into a Microsoft Access database. Data to be abstracted will include citation information, study years and locations, study design, methods and sites used to recruit study participants, method used to determine HCV infection and reinfection, types of interventions studied, sample size and demographic, and other characteristics of the study sample (e.g., mucosally traumatic sexual practices and sex under the influence of drugs).

Crude and adjusted measures of association (odds ratios, relative risks, hazard ratios, etc.) between disease outcomes (HCV infection and reinfection) and relevant exposures, confounding and moderating factors will be recorded. For those reports that are missing key data of interest to this systematic review but were thought potentially to have collected these data, the corresponding authors will be contacted.

### Quality assurance

Screening and data abstraction will be carried out by staff with graduate training in research methodology and additional training in HCV epidemiology, and systematic review and meta-analysis methods. A pilot study will be carried out to test and refine procedures for screening and data abstraction. Two staff will independently screen and code a subset of articles retrieved using the search criteria and will compare results. Discrepancies between the results will be discussed and the protocol will be revised to clarify procedures. This process will be repeated until consensus is reached. Reports that were deemed ineligible will be reviewed by the project director (AEJ) to ensure that no eligible reports are excluded. In addition, all coding will be reviewed for accuracy and completeness by the project director and the principal investigator (HH). Weekly staff meetings will discuss and resolve any issues that may arise. A written study manual will be developed to guide the process and to record special cases and their resolution.

### Study quality and critical appraisal

In this synthesis, bias in the included studies may arise in the form of selection bias, misclassification of exposure or outcome, and confounding due to non-comparability of the groups being compared. The quality rating procedure that will be employed is based on the Newcastle–Ottawa Scale, which assigns quality ratings to studies in relation to these threats to internal validity (selection bias, misclassification and non-comparability) [33]. Some types of bias will be addressed through screening of reports for eligibility. For example, we will exclude reports that may have misclassified HCV exposure (sexual vs injection related) by failing to exclude MSM who inject drugs from their analyses. Eligibility screening also will address potential misclassification of the outcome (e.g., acute or recent vs chronic HCV infection). In addition to the Newcastle–Ottawa Scale, publication bias will be examined by comparing mean effect sizes between published and unpublished studies and by the use of funnel plots [34].

#### *Selection bias*

The potential for selection bias in case–control studies will be evaluated in relation to whether similar and adequate methods were used to classify case vs control status, and whether cases and controls arose from the same underlying population (in other words, ‘If controls had acquired HCV infection or had progressed toward cirrhosis, would they appear as cases in this study?’). In cohort studies, selection bias will be judged in relation to whether ascertaining exposure or selection of the exposed cohort was related to likelihood of HCV infection, and whether methods were adequate in assuring that HCV infection was not present at the start of the cohort study (see European NEAT’s preferred criteria discussed above).

#### *Comparability*

Comparability of cases and controls refers to whether matching or adjustment for confounding (differences in the distribution of factors across cases and controls that could bias estimates of association) was carried out. In cohort studies, comparability refers to whether the assessment of the association between exposure and HCV infection adjusted for important differences between the exposed and unexposed cohorts.

#### *Misclassification*

In case–control studies, classification of cases and controls with respect to exposure must be unbiased, and using the same method to ascertain exposure for cases and controls is preferred. Ascertainment of exposure (factors related to sexual HCV transmission or progression) also relates to the extent to which reliable methods were used to determine that HIV-positive MSM were not also injection drug users. In cohort studies, misclassification of the outcome (e.g., acute or recent HCV infection) will be addressed as part of the eligibility screening. Studies using the European NEAT alternative criteria (described above [30]) will be given lower quality ratings.

### Data analysis

Synthesis will begin with the search for homogeneous subsets within sets of studies, followed by meta-analysis and calculation of summary estimates within the homogeneous subsets. Evidence of heterogeneity will be evaluated at each step in the analysis to distinguish between a true variation of effects and heterogeneity due to other differences. Effect measures reported as hazards ratios, risk ratios or relative risks will be transformed into odds ratios using standard methods [35]. Forest plots will also be used to assess underlying heterogeneity in relation to covariates and quality ratings [36-38].

Some statistical heterogeneity in a meta-analysis is inevitable due to the methodological diversity of the studies. We will use Cochran’s Q statistic [37] to determine whether observed heterogeneity is compatible with chance, and quantify variability using the *I*^2^ statistic [39]. We will take *I*^2^, the τ-statistic (variability due to inter-study variance), as well as methodological, study quality and other differences into consideration in determining whether to pool estimates. Effect size estimates will be combined using standard meta-analytic techniques in the form of pooled odds ratios and their 95% confidence intervals [40]. We will use random effects calculations whenever possible [41]. Potential moderator effects will be tested using meta-regression [42,43].

## Discussion

This systematic review and meta-analysis will critically assess the evidence related to sexual HCV transmission in a key affected population – HIV-positive MSM. It is anticipated that this review will identify modifiable behavioral risk factors and settings, which will be the target of interventions to control transmission. These, along with estimates of the range and magnitude of HCV transmission in this population, will be key inputs to simulations that will address the question of how to constitute a portfolio of interventions so as to deliver the greatest health benefit given a particular budget.

During the past decade, clusters of HCV infection in 15 to 24 year olds who have transitioned from abuse of prescription opioids to illicit opiate injection have been reported throughout the US [44,45]. As the epidemic of abuse of prescription opioids matures [46], it will likely serve as a persistent source of new injectors, and HCV incidence in new injectors may exceed 40/100 PY [47,48]. The parallel emergence of outbreaks of HCV infection in prescription opioid users and HIV-positive MSM raises important and urgent questions regarding how to allocate scarce public health resources to control HCV in the US. Early reviews reported the median HCV incidence in HIV-positive MSM was 6/1,000 PY [28]. Furthermore, HCV infection is hyperendemic in older people who inject or have ever injected drugs (PWID), with incidence rates between 10/100 PY to 40/100 PY throughout the world. The median prevalence in PWID in the US is estimated to be 65% [21,22]. Thus, an effective HCV control strategy must prioritize PWID. The HCV Synthesis Project will conduct a series of systematic reviews and meta-analyses to synthesize the evidence across all three of these population groups and, in collaboration with key community stakeholders, will develop recommendations and decision tools to guide public health policymakers toward an effective and feasible program of HCV control.

## Abbreviations

ALT: alanine aminotransferase; CI: confidence interval; HCV: hepatitis C virus; MSM: men who have sex with men; PWID: people who inject drugs; PY: person years.

## Competing interests

The authors declare that they have no competing interests.

## Authors’ contributions

HH conceived and designed the study and wrote the manuscript. JN collected data and tested the protocol. AEJ, DCDJ, JW, KD, BK, RSB and JK conceived and designed the study and undertook a critical revision of the manuscript. All authors have read and approved the final version of the manuscript.
